# Household transport ownership and HIV viral suppression in rural Uganda: a cross- sectional, population-based study

**DOI:** 10.21203/rs.3.rs-4288433/v1

**Published:** 2024-04-29

**Authors:** Joseph Ssuuna, Ping Teresa Yeh, Godfrey Kigozi, Fred Nalugoda, Gertrude Nakigozi, Joseph Kagaayi, Ronald M. Galiwango, Joseph G. Rosen, Steven J. Reynolds, Thomas C. Quinn, Maria J. Wawer, Ronald H. Gray, M. Kate Grabowski, Larry W. Chang

**Affiliations:** Rakai Health Sciences Program; Johns Hopkins Bloomberg School of Public Health; Rakai Health Sciences Program; Rakai Health Sciences Program; Rakai Health Sciences Program; Makerere University College of Health Sciences; Rakai Health Sciences Program; Johns Hopkins Bloomberg School of Public Health; Johns Hopkins School of Medicine; Johns Hopkins Bloomberg School of Public Health; Johns Hopkins Bloomberg School of Public Health; Johns Hopkins Bloomberg School of Public Health; Johns Hopkins School of Medicine; Johns Hopkins School of Medicine

**Keywords:** transportation, HIV care and treatment, viral load suppression, people living with HIV, Uganda, sub-Saharan Africa, population-based study

## Abstract

**Background:**

Empirical data on transportation access and HIV treatment outcomes in sub-Saharan Africa are rare. We assessed the association between household transport ownership and HIV viral suppression in rural Uganda.

**Methods:**

The study was conducted among people living with HIV aged 15–49 years using cross-sectional data from the Rakai Community Cohort Study (RCCS), collected from June 14, 2018, to November 6, 2020. Transport ownership was defined as household possession of a car, motorcycle, or bicycle. HIV viral suppression was defined as < 1000 HIV RNA copies/ml. Poisson regression with robust variance estimation identified unadjusted and adjusted prevalence ratios and 95% confidence intervals (CI) of HIV viral suppression by transport ownership.

**Results:**

The study included 3,060 persons aged 15–49 living with HIV. Overall HIV viral suppression was 86.5% and was higher among women compared to men (89.3% versus 81.6%; adjusted prevalence ratio: 1.14, 95% CI: 1.10, 1.18). A total of 874 participants (28.6%) resided in households that owned at least one means of transport. HIV viral suppression was 79.8% among men and 88.2% among women from households without any means of transport, compared to 85.4% among men and 92.4% among women from households with at least one means of transport. Adjusted prevalence ratios of HIV viral suppression were 1.11 (95% CI: 1.04, 1.18) for males and 1.06 (95% CI: 1.03, 1.10) for females from households owning at least one means of transport compared with those from households with none.

**Conclusion:**

There was increased HIV viral suppression among people living with HIV from households with transport means compared to those from households without transport means, suggesting transport may facilitate access to, and continued engagement with, HIV treatment services.

## Background

Sub-Saharan Africa has the highest burden of HIV globally,^1^ and achieving HIV viral suppression among people living with HIV reduces mortality and onward transmission.^2^ However, despite HIV treatment scale-up, many people living with HIV remain virally unsuppressed.^3^ For example, one study in south-central Uganda reported a viral suppression rate of 72.4% at the end of 2022;^4^ another study conducted in south-western Uganda among adolescents found 81% viral suppression.^5^ This threatens to stall progress in the HIV response towards the UNAIDS 95-95-95 HIV treatment targets by 2030.^6^

Multiple studies, including a systematic review of studies in sub-Saharan Africa, have highlighted absence of transport as a potential barrier to antiretroviral therapy adherence.^7–9^ Additionally, travel costs and distance from households to HIV clinics have been identified as constraints to accessing HIV care.^10^ In Uganda, salient barriers to accessing healthcare include distance from households to clinics, perceived quality of care, and drug availability.^11^ A qualitative study conducted with caregivers of children living with HIV in western Uganda showed that transport was a major barrier among participants exhibiting viremia.^12^ Costs incurred traveling to HIV clinics and difficulty finding HIV clinics within reasonable travel distances from the home were shown to impede HIV care access individuals who were virologically unsuppressed in another study in rural Uganda.^13^

Few studies have empirically explored the relationship between household transport ownership and clinical HIV outcomes. A mathematical model in Malawi singled out limited ownership of cars, motorcycles, and bicycles as a driver of HIV treatment disengagement.^14^ By comparison, a study among pregnant women in four African countries found no association between owning any means of transport and completion of the HIV prevention of mother to child transmission treatment cascade.^15^ Given the lack of empirical data on this relationship, we assessed the association between household transport ownership (car, motorcycle, or bicycle) and HIV viral suppression in rural Uganda, leveraging data from a large, population-based HIV surveillance cohort.

## Methods

### Study design and population.

This cross-sectional study used data from the Rakai Community Cohort Study (RCCS)—an open, prospective, population-based HIV surveillance cohort that enrolls consenting persons aged 15–49 years. The RCCS is implemented in 40 communities in rural south-central Uganda, which are broadly categorized as semi-urban trading centers, agrarian villages, and fishing communities^16^ with varying HIV burdens.^17^ A household census is conducted followed by survey interviews for eligible household members.^16^ This study was conducted using data from RCCS participants living with HIV, confirmed by laboratory results in survey round 19 (implemented from June 14, 2018 to November 6, 2020).

Transport ownership (car, motorcycle, or bicycle) was assessed through the household census, which asked the head of household about household possessions including motor vehicles and bicycles. Household transport ownership was enumerated from self-reported possession of at least one of the three means of transport. Blood samples were collected from all consenting RCCS participants, and viral load testing was completed for those with serologically confirmed HIV infection. HIV viral load testing was performed using Abbott m2000sp and m2000rt combined system. HIV viral suppression was defined as < 1000 HIV RNA copies/ml.^18^

### Statistical analysis

The main outcome of interest was HIV viral suppression, measured dichotomously: suppressed or unsuppressed. The primary independent variable was household transport ownership, measured categorically (no ownership, bicycle, motorcycle, car, and at least one means of transport). Other variables considered included sex, age (with a spline at 40 years), community type (agrarian, trading, and fishing), highest level of education attended (no formal education, primary, secondary, and tertiary), recent migration (in or within communities), socio-economic status (using Santelli et al.’s composite variable of enumerated household assets)^19^, distance (in kilometers) from household to the closest antiretroviral therapy (ART) dispensing facility (measured using a geodesic distance calculation from GPS coordinates of households and health facilities in the RCCS surveillance area, dichotomized to < 5 kilometers versus > = 5 kilometers based on the reasoning that there may be fewer transportation-related barriers to accessing health services in households closer to health facilities), and status as head of the household.

Poisson regression with robust variance estimation was used to identify unadjusted and adjusted prevalence ratios (adjPR) and 95% confidence intervals (CI). A priori, age and sex were considered potential confounders of the relationship between household transport ownership and viral suppression. Akaike’s Information Criterion (lowest AIC) was used to select a parsimonious multivariable model adjusting for sex, age, community type, education level, distance from home to nearby health facility, and recent migration status. Analyses were further stratified by sex, head of household status, and SES. Data analysis was conducted using Stata/BE 17.1 (StataCorp LLC, College Station, Texas).

## Results

There were 3,332 individuals living with HIV, most of whom (64.0%) were female. Thirty participants (0.9%) had missing data on the primary exposure of interest (household transport ownership), and 242 participants were missing data on distance from home to nearest facility because they were missing global positioning system (GPS) coordinates for their homes; these were excluded from the primary analysis. Table 1 shows sociodemographic characteristics of the 3,060 participants living with HIV aged 15–49 years included in the analysis. A total of 874 participants (28.6%) resided in households that owned at least one means of transport. Most people with household transport lived in homes with a bicycle (20.8%, n = 637), and fewer with a motorcycle (12.2%, n = 372) or car (2.5%, n = 77). The mean age of all participants was 35.7 (standard deviation 7.5) years. The mean age was comparable between those whose households owned at least one means of transport and those that did not own any. Almost half of the participants (44.1%, n = 1,350/3,060) resided in households that were more than five kilometers from the nearest health facility. The overall mean distance from households to the closest facility was 5.0 (standard deviation 3.1) kilometers (Supplemental Fig. 1).

**Figure 1:**
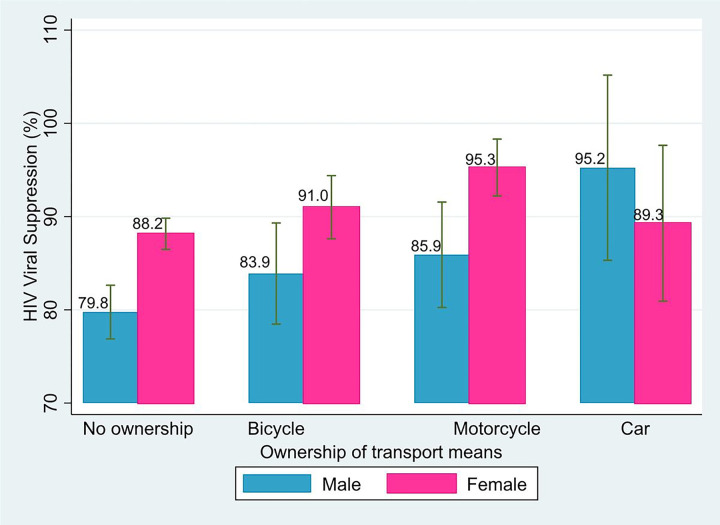
HIV viral suppression rates by transport ownership for 3,060 people living with HIV

Most participants (86.5%, n = 2,647/3,060) were virally suppressed, including 81.6% of males (n = 898/1,101) and 89.3% of females (n = 1,749/1,959). Overall, viral suppression was 85.3% (n = 1,864/2,186) for those who resided in households that did not own any means of transport, versus 89.6% (n = 783/874) for those owning at least one means of transport. Viral suppression was 88.2% (n = 404/458) for those from households with bicycles, 91.2% (n = 309/339) for those from households with motorcycles, and 90.9% (n = 70/77) for those from households with cars. Among males, all forms of household transport ownership were associated with higher levels of viral suppression; conversely, only bicycle and motorcycle ownership were associated with higher levels of viral suppression among females (Fig. 1).

**Figure 2:**
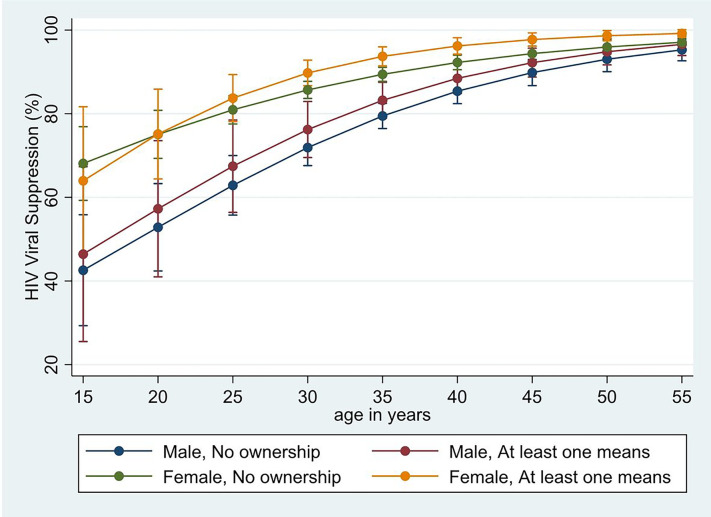
HIV viral suppression by age and sex for 3,060 people living with HIV stratified by transport ownership.

Figure 2 illustrates the non-linear relationship between HIV viral suppression and age, stratified by means of transport ownership and sex. Among males, viral suppression was consistently higher in individuals who resided in households with any transport ownership than in households without transport ownership. However, among females, differences in viral suppression by household transport ownership were only observed in older age groups and generally smaller than those observed in men.

Table 2 presents associations between household transport ownership and HIV viral suppression—overall and by population strata. After adjusting for sex, age, level of education, distance from household to nearest health facility, and community type, the prevalence ratio of HIV viral suppression was significantly higher (adjPR: 1.08, 95% CI: 1.05, 1.11) among participants who resided in households with transport ownership compared to those without transport ownership. Results were similar irrespective of sex (men: adjPR: 1.11, 95% CI: 1.04, 1.18; women: adjPR: 1.06, 95% CI: 1.03, 1.10). In analyses stratified by SES, transport ownership had a larger effect on viral suppression among persons from households with lower and middle SES, respectively, compared to individuals with higher SES, and among persons residing further from the closest health facility.

## Discussion

HIV viral suppression was approximately 8% higher among individuals from households that owned at least one means of transport compared to individuals from households that did not own any means of transport. The percentage increase of viral suppression, comparing any transport ownership to none, was larger among men than women (11% versus 6%). These findings are consistent with the results of the modeling study in Malawi that found limited transport ownership as a barrier to HIV treatment coverage and viral suppression, and recommended increasing availability of bicycles in rural areas.^14^

Household transport ownership may facilitate increased access and engagement with HIV services. Additionally, individuals in household with transportation access may have greater autonomy to seek HIV services from facilities with perceived better quality of care or where they are less likely to experience stigma, which have been identified as issues affecting access and utilization of health services in a systematic review of Ugandan health services.^11^ Billioux et al found that in the Rakai region, 57% of individuals living with HIV sought care from health facilities further away from their homes compared to their closest ART dispensing venue,^20^ which could be an indicator of facility preference enabled by household transport ownership.

Our findings suggest that there is need to explore how household transport ownership can be increased from the current relatively low 28.6% and whether such assets would impact HIV-related outcomes. Transport interventions – whether increasing transport ownership, increasing access to transportation resources, or expanding community-based differentiated service delivery models – may contribute towards improved HIV viral suppression from current rates of ~ 75–95% targets by 2030.^3^ In particular, more programmatic focus should be placed on men, who showed a stronger association between HIV viral suppression and household transport ownership. Prior data from this same cohort in south-central Uganda showed that limited HIV viral suppression among men was a major driver of continued HIV transmission in the region.^21^ Increasing viral load suppression – simply by providing bicycles – among men could potentially attenuate gendered disparities in HIV viral suppression.

Strengths of this study included a large sample size from a well-established prospective HIV surveillance cohort. Additionally, to our knowledge, this is the first quantitative study of the association of household transport ownership and HIV viral suppression in sub-Saharan Africa. This study also had several limitations. For example, we assumed that any member of the household could use the means of transport that was available at home to travel to the facility for HIV services. Furthermore, we did not know the mechanism through which participants were receiving HIV care, e.g., some individuals may have received care through community-based differentiated service delivery models, which could have rendered them less reliant on transport. We also were unable to distinguish between specific types of transport and their association with viral suppression. Finally, because of the cross-sectional study design, we could not ascertain if changes in transport ownership were prospectively associated with improved HIV viral suppression.

## Conclusions

There was a significant increase in HIV viral suppression among persons living with HIV who resided in households that owned a means of transport compared to those who did not. Increasing household transport ownership may contribute towards HIV viral suppression targets, particularly for men, and should be explored as an HIV care intervention.

## Figures and Tables

**Table 1:** Socio-demographic characteristics of 3,060 participants by household transport ownership.

Number (Percentage)	Bicycle ownership		Motorcycle ownership		Car ownership		Ownership of at least one means of transport		Total
	Yes	p-value	Yes	p-value	Yes	p-value	Yes	p-value	
Overall (%)	637 (20.8)		372 (12.2)		77 (2.5)		874 (28.6)		3060
Age: mean (SD)	37.2 (7.7)		36.8 (7.8)		37.6 (8.4)		36.9 (7.7)		35.7 (7.5)
**Sex**									
Male	252 (20.8)	0.034	159 (14.4)	0.004	21 (1.9)	0.107	350 (31.8)	0.003	1101 (36.0)
Female	385 (19.7)		213 (10.9)		56 (2.9)		524 (26.8)		1959 (64.0)
**Education level**									
No formal education	30 (11.9)	0.001	11 (4.4)	< 0.001	2 (0.8)	< 0.001	37 (14.6)	< 0.001	253 (8.3)
Primary	450 (20.8)		248 (11.4)		42 (1.9)		607 (28.0)		2169 (70.9)
Secondary	138 (24.4)		96 (17.0)		28 (5.0)		201 (35.5)		566 (18.5)
Tertiary	18 (25.7)		17 (24.3)		7 (7.1)		28 (40.0)		70 (2.3)
**Marital status**									
Single	130 (13.2)	< 0.001	31 (3.14)	< 0.001	13 (1.3)	0.006	158 (16.0)	< 0.001	986 (32.2)
Married	458 (24.5)		325 (17.4)		60 (3.2)		663 (35.5)		1869 (61.1)
**Head of household**									
Not head of household	317 (26.1)	< 0.001	200 (16.5)	< 0.001	48 (4.0)	< 0.001	439 (36.2)	< 0.001	1214 (39.7)
Head of household	320 (17.3)		172 (9.3)		29 (1.6)		435 (23.6)		1846 (60.3)
**Community type**									
Agrarian	340 (33.1)	< 0.001	168 (16.4)	< 0.001	25 (2.4)	< 0.001	428 (41.7)	< 0.001	1027 (33.6)
Fishing	119 (8.4)		96 (6.8)		20 (1.4)		194 (13.6)		1423 (46.5)
Trading	178 (29.2)		108 (17.7)		32 (5.3)		252 (41.3)		610 (19.9)
**SES**									
Low	100 (8.6)	< 0.001	44 (3.8)	< 0.001	(0.1)	< 0.001	132 (11.3)	< 0.001	1165 (38.1)
Middle	144 (15.1)		79 (8.3)		6 (0.6)		212 (22.2)		954 (31.2)
High	390 (41.6)		247 (26.4)		70 (7.5)		527 (56.2)		937 (30.7)
**Distance from home to nearest health facility**									
<5km	428 (25.0)	< 0.001	254 (14.9)	< 0.001	52 (3.0)	0.037	584 (34.2)	< 0.001	1710 (55.9)
≥5km	209 (15.5)		118 (8.7)		25 (1.9)		290 (21.5)		1350 (44.1)
Mean distance (SD)	4.3 (2.7)		4.2 (2.8)		4.7 (2.9)		4.4 (2.7)		5.0 (3.1)
**Migration status**									
Non migrant	531 (22.8)	< 0.001	309 (13.3)	< 0.001	66 (2.8)	0.043	720 (31.0)	< 0.001	2326 (76.0)
Migrants (in/within community)	106 (14.4)		63 (8.6)		11 (1.5)		154 (21.0)		734 (24.0)

**Table 2:** Association between HIV viral suppression and ownership of means of transport for 3,060 people living with HIV, stratified by demographics and distance from home to facility.

		Household transport ownership	Total (N)	HIV viral suppression n (%)	Crude prevalence ratio (95% CI)	p-value	Adjusted prevalence ratio (95% CI)	p-value
Overall*		No ownership	2186	1864 (85.3)	Ref		Ref	
		Any transport	874	783 (89.6)	1.05 (1.02, 1.08)	< 0.001	1.08 (1.05, 1.11)	< 0.001
		Bicycle	458	404 (88.2)	1.03 (1.00, 1.07)	0.08	1.06 (1.02, 1.10)	< 0.001
		Motorcycle	339	309 (91.2)	1.07 (1.03,1.11)	<0.001	1.10 (1.06, 1.15)	< 0.001
		Car	77	70 (90.9)	1.07 (0.99,1.15)	0.09	1.08 (1.01, 1.16)	0.04
Sex	Men	No ownership	751	599 (79.8)	Ref		Ref	
		Any transport	350	299 (85.4)	1.07 (1.01, 1.13)	0.02	1.11 (1.04, 1.18)	< 0.001
		Bicycle	180	151 (83.9)	1.05 (0.98, 1.13)	0.19	1.09 (1.00, 1.18)	0.04
		Motorcycle	149	128 (85.9)	1.08 (1.00, 1.13)	0.05	1.11 (1.03, 1.20)	< 0.001
		Car	21	20 (95.2)	1.19 (1.07, 1.32)	< 0.001	1.20 (1.07, 1.35)	< 0.001
	Women	No ownership	1435	1265 (88.2)	Ref		Ref	
		Any transport	524	484 (92.4)	1.05 (1.02, 1.08)	0.02	1.06 (1.03, 1.10)	< 0.001
		Bicycle	278	253 (91.0)	1.03 (0.99, 1.08)	0.13	1.04 (1.00, 1.09)	0.04
		Motorcycle	190	181 (95.3)	1.08 (1.04, 1.12)	< 0.001	1.10 (1.06, 1.14)	< 0.001
		Car	56	50 (89.3)	1.01 (0.92, 1.11)	0.79	1.04 (0.95, 1.13)	0.44
Household head	Head of the household	No ownership	1411	1205 (85.4)	Ref		Ref	
		Any transport	435	383 (88.0)	1.03 (0.99, 1.07)	0.19	1.06 (1.01, 1.11)	1.01
		Bicycle	246	216 (87.8)	1.03 (0.98, 1.08)	0.29	1.04 (0.99, 1.10)	0.13
		Motorcycle	160	140 (87.5)	1.03 (0.96, 1.08)	0.45	1.07 (1.01, 1.14)	0.03
		Car	29	27 (93.1)	1.09 (0.99, 1.21)	0.10	1.09 (0.97, 1.22)	.013
	Not head of household	No ownership	775	659 (85.0)	Ref		Ref	
		Any transport	439	400 (91.1)	1.07 (1.03, 1.12)	< 0.001	1.08 (1.03, 1.14)	0.01
		Bicycle	212	188 (88.7)	1.04 (0.99, 1.10)	0.15	1.06 (1.00, 1.12)	0.06
		Motorcycle	179	169 (94.4)	1.11 (1.06, 1.16)	< 0.001	1.12 (1.06, 1.18)	< 0.001
		Car	48	43 (89.6)	1.05 (0.98, 1.17)	0.21	1.06 (0.97, 1.17)	0.28
Distance from home to nearest ART dispensing facility	< 5km	No ownership	1126	953 (84.6)	Ref		Ref	
		Any transport	584	515 (88.2)	1.04 (1.00, 1.08)	0.04	1.07 (1.03, 1.11)	< 0.001
		Bicycle	301	260 (86.4)	1.02 (0.97, 1.07)	0.44	1.04 (0.99, 1.10)	0.10
		Motorcycle	231	208 (90.0)	1.06 (1.01, 1.12)	0.01	1.10 (1.05, 1.15)	< 0.001
		Car	52	47 (90.4)	1.06 (0.97, 1.17)	0.16	1.10 (1.01, 1.21)	0.03
	≥ 5km	No ownership	1060	911 (85.9)	Ref		Ref	
		Any transport	290	268 (92.4)	1.08 (1.03, 1.12)	< 0.001	1.09 (1.04, 1.14)	< 0.001
		Bicycle	157	144 (91.7)	1.07 (1.01, 1.13)	0.02	1.08 (1.02, 1.15)	0.01
		Motorcycle	108	101 (93.5)	1.09 (1.03, 1.15)	< 0.001	1.11 (1.05, 1.19)	< 0.001
		Car	25	23 (92.0)	1.07 (0.95, 1.20)	0.23	1.04 (0.92, 1.18)	0.49
SES	Low	No ownership	1033	897 (86.8)	Ref		Ref	
		Any transport	132	120 (90.9.5)	1.05 (0.99, 1.11)	0.13	1.07 (1.01, 1.13)	0.03
		Bicycle	87	77 (88.5)	1.02 (0.947, 1.11)	0.64	1.05 (0.97, 1.13)	0.23
		Motorcycle	44	42 (95.5)	1.10 (1.03, 1.18)	0.01	1.10 (1.02, 1.18)	0.01
		Car	1	1 (100)	1.15 (1.12, 1.18)	< 0.001	1.18 (1.11, 1.26)	< 0.001
	Middle	No ownership	742	610 (82.2)	Ref		Ref	
		Any transport	212	186 (87.7)	1.07 (1.01, 1.13)	0.04	1.08 (1.02, 1.15)	0.01
		Bicycle	1278	110 (86.6)	1.05 (0.98, 1.14)	0.18	1.07 (0.99, 1.16)	0.09
		Motorcycle	79	70 (88.6)	1.08 (0.99, 1.18)	0.09	1.10 (1.01, 1.19)	0.03
		Car	6	6 (100)	1.22 (1.18, 1.26)	< 0.001	1.26 (1.15, 1.39)	< 0.001
	High	No ownership	410	356 (86.8)	Ref		Ref	
		Any transport	527	474 (89.9)	1.04 (0.99, 1.09)	0.14	1.04 (0.99, 1.09)	0.13
		Bicycle	243	216 (88.9)	1.02 (0.97, 1.09)	0.43	1.02 (0.96, 1.09)	0.47
		Motorcycle	214	195 (91.1)	1.05 (0.99, 1.11)	0.09	1.06 (1.00, 1.13)	0.04
		Car	70	63 (90.0)	1.04 (0.95, 1.13)	0.42	1.03 (0.95, 1.13)	0.44

* Adjusted for sex, age, community type, education level, distance from home to nearby health facility, and recent migration status

## Data Availability

The datasets analyzed during the current study are available from the corresponding author upon reasonable request.

## Abbreviations

adjPR: adjusted prevalence ratio
ART: Anti-Retroviral Therapy
CI: confidence interval
HIV: Human Immunodeficiency Virus
RCCS: Rakai Community Cohort Study
RHSP: Rakai Health Sciences Program
UNAIDS: Joint United Nations Program on HIV/AIDS
